# Medical marijuana users in substance abuse treatment

**DOI:** 10.1186/1477-7517-7-3

**Published:** 2010-03-05

**Authors:** Ronald Swartz

**Affiliations:** 1 Department of Social Work, Humboldt State University, Arcata, CA 95521, USA

## Abstract

**Background:**

The rise of authorized marijuana use in the U.S. means that many individuals are using cannabis as they concurrently engage in other forms of treatment, such as substance abuse counseling and psychotherapy. Clinical and legal decisions may be influenced by findings that suggest marijuana use during treatment serves as an obstacle to treatment success, compromises treatment integrity, or increases the prevalence or severity of relapse. In this paper, the author reviews the relationship between authorized marijuana use and substance abuse treatment utilizing data from a preliminary pilot study that, for the first time, uses a systematic methodology to collect data examining possible effects on treatment.

**Methods:**

Data from the California Outcomes Measurement System (CalOMS) were compared for medical (authorized) marijuana users and non-marijuana users who were admitted to a public substance abuse treatment program in California. Behavioral and social treatment outcomes recorded by clinical staff at discharge and reported to the California Department of Alcohol and Drug Programs were assessed for both groups, which included a sample of 18 reported medical marijuana users.

**Results:**

While the findings described here are preliminary and very limited due to the small sample size, the study demonstrates that questions about the relationship between medical marijuana use and involvement in drug treatment can be systematically evaluated. In this small sample, cannabis use did not seem to compromise substance abuse treatment amongst the medical marijuana using group, who (based on these preliminary data) fared equal to or better than non-medical marijuana users in several important outcome categories (e.g., treatment completion, criminal justice involvement, medical concerns).

**Conclusions:**

This exploratory study suggests that medical marijuana is consistent with participation in other forms of drug treatment and may not adversely affect positive treatment outcomes. These findings call for more extensive sampling in future research to allow for more rigorous research on the growing population of medical marijuana users and non-marijuana users who are engaged in substance abuse treatment.

## Background

A natural experiment is unfolding in California related to the therapeutic use of marijuana as a component of substance abuse treatment for alcohol, methamphetamine, heroin, cocaine, and other drugs of abuse. For up to 13 years now, people have been authorized to use marijuana for recognized medical purposes under California's "Compassionate Use Act of 1996" (also known as Prop. 215) and, more recently, the "Medical Marijuana Program" (also known as SB 420). Despite U.S. Drug Enforcement Administration refusal to recognize any legitimate medical use of marijuana, counties in California remain accountable to the state government for implementation of medical marijuana laws as passed by voter initiative and legislative action. Most county governments and public social service programs in the state have recognized the legal right of qualified patients to use marijuana in a variety of settings. For some public agencies that provide substance abuse treatment this has included authorization to use marijuana during the course of treatment.

Several studies have linked cannabis to psychosis, schizophrenia, anxiety, depression and other adverse physical, psychological, and social outcomes [1-6]. Meanwhile, marijuana's positive therapeutic effects have been documented by the U.S. Institute of Medicine [7], the American College of Physicians [8], and other sources [9-12] in relation to psychosis, bipolar disorder, anxiety, pain, anorexia, nausea, and muscle spasticity, including randomized, placebo-controlled, crossover trials [13]. Despite Macleod's [14] analysis of weaknesses in methodology, analysis, and interpretation of studies linking marijuana use to mental illness, Hall & Room's [15] comprehensive review of published studies concluded that there is reasonable evidence to suggest that regular cannabis use predicts an increased risk of schizophrenia and psychotic symptoms. They also concluded that the public health harm from cannabis remains substantially lower than from legal substances such as alcohol and tobacco. Consistent answers to questions about marijuana's social and health effects still allude clinical, scholarly, and legal domains.

Noticeably missing from research literature related to marijuana's therapeutic potential is any examination of its influence on substance abuse treatment outcomes. Decades old studies on the therapeutic effects of psychedelics on alcoholism are generally regarded with a bit of suspicion, though some researchers attest to their veracity [16]. The National Institute on Drug Abuse sponsored studies in the early 1990s related to ibogaine, the active ingredient in the African iboga plant, for treatment of cocaine addiction [17]. In 2002, the FDA approved research on MDMA for PTSD treatment [18]. Reiman's [19] surveys of medical marijuana consumers at cannabis dispensaries in the San Francisco Bay area demonstrated nearly half of respondents had "substitu [ed] cannabis for alcohol and illegal drugs," including 74% who reported using marijuana instead of prescription drugs (p. 31).

Experimental research on marijuana's role in addiction treatment presents legal and political difficulties. Even as the American College of Physicians recommends "programs and funding for rigorous scientific evaluation of the potential therapeutic benefits of medical marijuana and the publication of such findings" ([8], p. 3), clinical trials that would administer marijuana to people undergoing substance abuse treatment are highly unlikely to receive approval. Therefore, any examination of marijuana's effect on treatment outcomes must necessarily make use of existing data.

Demonstrating the impact of marijuana use on treatment outcomes is important for developing an expansive evidence-base for treatment alternatives. Examining the negative, positive, or neutral consequences of marijuana use is also critical for evaluating abstinence-only and harm reduction models for addiction treatment. Harmful social, psychological, and behavioral effects of marijuana use pale in comparison to other common drugs of abuse, including alcohol, methamphetamine, cocaine, and heroin [20]. Clear evidence that people who enter substance abuse treatment for problematic use of more harmful drugs can exhibit equal or better outcomes when using marijuana during their treatment offers a compelling case for further research on this particular dimension of marijuana as medicine. Findings that suggest marijuana use during treatment serves as an obstacle to treatment, compromises treatment integrity, or increases the prevalence or severity of relapse may similarly influence legal and clinical decisions.

Just as legal pharmaceutical substances are routinely prescribed in the course of substance abuse treatment, marijuana may provide a less harmful alternative to the drug problems bringing people in. Substitution of a lower risk psychoactive substance for a more harmful psychoactive substance has been regarded as legitimate clinical practice for at least half a decade [21]. Clinical communities will be impacted by findings as they will need to develop proper treatment protocols for current medical marijuana users. Treatment outcomes of authorized marijuana users may suggest that use of marijuana instead of riskier substances is an important step toward abstinence for some treatment clients, while it may serve as a long-term solution for others, much like pharmaceutical opiates such as methadone and buprenorphine [21]. Contrarily, studies that reveal poorer outcomes for medical marijuana users in treatment may push public agencies to revise protocols that currently allow for continued medical use of cannabis during substance abuse treatment. The aim of the study presented here was to demonstrate an ethical and legitimate methodology for engaging in such research.

## Methods

Data was collected from a nonprobability, convenience sample composed of all clients in one California county identified as authorized medical marijuana users who were admitted to substance abuse treatment during the study time period. The comparison group consisted of all other county treatment clients with similar admission dates and primary drug use reported. While this could be considered a quasi-experimental comparison group design with no controlled randomization and a limited sample size, the study is best described as an exploratory pilot investigation due to the final sample size.

All publicly funded substance abuse treatment agencies in California must report admission and discharge data to the State Department of Alcohol and Drug Programs via the California Outcomes Measurement System (CalOMS). While CalOMS has some limitations, noticeably in relation to specificity of treatment outcomes, it is the best available database for cohort comparisons across a range of domains. Access to county level CalOMS data was provided by the participating county as were specific data points for those clients certified as medical marijuana users (no personally identifiable data were presented to the researcher).

The Attorney General of California has defined a qualified medical marijuana patient as someone "whose physician has recommended the use of marijuana to treat a serious illness, including cancer, anorexia, AIDS, chronic pain, spasticity, glaucoma, arthritis, migraine, or any other illness for which marijuana provides relief"([22], p. 4). The Attorney General's guidelines also note that "criminal defendants and probationers may request court approval to use medical marijuana while they are released on bail or probation" (p. 6). Each of the medical marijuana users in this study was referred to substance abuse treatment by the criminal court, sought permission to use medical marijuana during treatment, and received such authorization.

Treatment staff identified 18 clients as medical marijuana users engaged in treatment at the beginning of the study. Staff were aware of clients' medical marijuana use and had documented it in clients' files. While the identities of these clients were never shared with the researcher, they were confirmed by multiple staff on repeated occasions. Though this substantially weakens the study's sampling protocol, no other option is currently available. Existing substance abuse treatment data systems do not record the status of a client as a medical marijuana user, so there is no independent way to establish who is a medical marijuana user in treatment and who is not other than through multiple substantiations from program staff. Of the initial set of 18 one died during the course of treatment and was excluded. Cause of death could not be determined from data collected, as client data files were not included in the study. Similarly, specific diagnoses could not be ascertained. In order to strengthen the research design, only those clients receiving outpatient drug free treatment in the county's substance abuse treatment program were included ("drug free" means they did not participate in an opiate maintenance or titration program). This resulted in the exclusion of one residential treatment client and three day treatment clients, leaving an experimental group size of 13. While including the day treatment and residential treatment clients would have increased the sample size, the significant variation in treatment protocols weakened the comparison to non-medical marijuana users. Admission dates for the 13 medical marijuana using clients were used as the basis for generating comparative data. Since they all indicated marijuana or methamphetamine as their primary drug of choice, the comparison group was limited to those treatment admissions where marijuana or methamphetamine was noted as the primary drug. In order to generate the comparison data set, county level reports on all adult treatment admissions between July 3, 2006 and November 16, 2007 (the admission dates for the medical marijuana group) who received Outpatient Drug Free treatment from the same treatment programs as the medical marijuana group, and who indicated marijuana or methamphetamine as their primary drug used were generated using CalOMS. These constitute separate reports in CalOMS, so *n*s were combined. From this combined data set, the *n*s for the medical marijuana group were subtracted leaving a comparison group composed of non-medical marijuana using treatment clients admitted in the same time period, receiving the same treatment services, and using the same primary drugs.

## Results

Table 1 presents major characteristics of the group of clients authorized to use marijuana during the course of substance abuse treatment (MM). As noted, the only group for which complete outcome data is available is the group that successfully completed treatment (*n *= 8). Though clinicians vary in their recommendations for the optimal length of treatment, it is generally accepted that the longer clients are engaged in treatment, the better their outcomes [23]. A very high percentage of the MM group who received at least four months of treatment completed or were discharged successful (80% [*n *= 8]), with a mean length for those completing treatment of five months, 8.4 days.

**Table 1:** Medical Marijuana (MM) Client Characteristics

Characteristic		%	*n*	Notes
**Age at admission**	18-25	23.1%	3	
	26-35	23.1%	3	
	36-45	38.5%	5	
	45<	15.4%	2	
**Gender**	Male	84.6%	11	
	Female	15.4%	2	
**Completed tx or made satisfactory progress at discharge**		69.2%	9	
**Completed tx**		61.5%	8	This is the only group for which complete outcome data is available
**Unsatisfactory discharge**		30.8%	4	
**Received at least 4 months of tx**		76.9%	10	
**Waitlist before admission**		15.4%	2	Mean of 5.77 days waited (Range = 0-45)
**Prior tx episodes**	0	53.8%	7	Mean of 1 prior tx episode (Range = 0-5) Mean of 1.86 (Range = 1-5) amongst those with prior tx episode(s)
	1	30.8%	4	
	2	15.4%	2	
	3<	7.7%	1	
**Race**	White	84.6%	11	
	American Indian	15.4%	2	All Non-white clients identified as American Indian
**Disability**^a^	Yes	66.7%	8	
	No	33.3%	4	
**Disabilities reported**^a^	Mental	37.5%	3	Mean of 1.73 disability categories per person
	Mobility	37.5%	3	
	Visual	37.5%	3	
	Hearing	12.5%	1	
	Speech	12.5%	1	
	Other	25.0%	2	
**Primary drug at admission**	Methamphetamine	38.5%	5	Mean use of 13.3 days in last month (Range = 0-30; Median = 10)
	Marijuana	61.5%	8	
**Age of 1st use of Primary drug**	12-14	30.8%	4	Mean of 16.4 years old (Range = 12-25)
	15-17	46.2%	6	
	18-20	7.7%	1	
	21<	15.4%	2	
**Primary or Secondary drug at admission**	No 2nd drug	7.7%	1	
	Alcohol	7.7%	1	
	Methamphetamine	84.6%	11	
	Marijuana	100.0%	13	
**Alcohol use in last 30 days at admission (alcohol is not Primary or Secondary)**		33.3%	4	Mean of .75 drinks/day in last month
**Needle use in last year**		7.7%	1	

a = Declined To State, Not Sure, and/or Don't Know not included

The catchment area for the public substance abuse treatment agency is not particularly ethnically diverse. The ethnic breakdown of the MM group (84.6% White, 15.4% American Indian) reflects disproportionate involvement of Native Americans in treatment. However, Native Americans represent one of the largest non-White populations in the region. Native Americans composed 10.3% of the control group. White clients were 71.9%.

Sixty-six point seven percent (*n *= 8) of the MM group reported having a disability and each person could indicate multiple disabilities. The most common disabilities reported include Mental, Mobility, and Visual.

Because 92% (*n *= 12) of the MM group reported poly-drug use at admission, Table 1 also presents the percentage of clients reporting use of alcohol, methamphetamine, *or *marijuana. While 38.5% (*n *= 5) of the MM group indicated primary use of methamphetamine at admission, 84.6% (*n *= 11) reported primary or secondary use of methamphetamine. Sixty-one point five percent (*n *= 8) of the MM group indicated primary use of marijuana at admission and 100% (*n *= 13) of clients in the MM group reported primary or secondary use of marijuana. Five people in the MM group indicated use of alcohol in the last 30 days, including four people for whom alcohol was not noted as a primary or secondary drug of use at admission.

Table 2 presents complete admission data for the 13 MM clients included in the data set, as well as the subset of clients who successfully completed treatment. Client outcomes are presented only for those who successfully completed treatment. Amongst those successfully completing treatment, use of all drugs other than marijuana had ceased in the month before discharge. This represents a drop in alcohol use from 50% (*n *= 4) to zero amongst those completing treatment when all clients who reported alcohol use at admission (primary, secondary, or other) are included. Of the 38.5% (*n *= 5) reporting methamphetamine use at admission, none reported methamphetamine use in the 30 days before discharge. Mean days of primary drug use in the last month stayed roughly the same at 16.1 (from 16.3). One client who reported needle use in the last year did not report intravenous drug use in the last 30 days.

**Table 2:** Medical Marijuana (MM) Client Outcomes

	Admission - All		Admission-Completers		Discharge-Completers	
	
	%	*n*	%	*n*	%	*n*
**Alcohol use in last 30 days (alcohol is not Primary or Secondary)**	33.3%	4	42.9%	3	0.0%	0
**Needle use in last year**	7.7%	1	12.5%	1	0.0%	0
**IV drug use in last 30 days**	0.0%	0	0.0%	0	0.0%	0
**Employed (includes FT & PT, excludes NILF)**	54.5%	6	50.0%	3	50.0%	3
**Worked in last 30 days**	33.3%^a^	4	28.6%	2	37.5%	3
**Currently enrolled in school**	7.7%	1	0.0%	0	12.5%	1
**Currently enrolled in job training**	0.0%	0	0.0%	0	12.5%	1
**Any criminal justice involvement in last 30 days**	30.8%	4	37.5%	3	0.0%	0
**Arrested in last 30 days**	15.4%	2	12.5%	1	0.0%	0
**Jailed in last 30 days**	15.4%	2	12.5%	1	0.0%	0
**Prison sentence in last 30 days**	7.7%	1	12.5%	1	0.0%	0
**ER visit in last 30 days**	23.1%	3	12.5%	1	0.0%	0
**Hospitalized in last 30 days**	15.4%	2	12.5%	1	0.0%	0
**Medical problems reported in last 30 days**	38.5%	5	37.5%	3	12.5%	1
**MH diagnosis**	45.5%^a^	5	50.0%	3	50.0%	3
**MH medication**	38.5%	5	37.5%	3	25.0%	2

a = Declined To State, Not Sure, and/or Don't Know not included

In relation to social outcomes, one client went from not looking for employment to "not in the labor force." Though one client went from looking for employment to not looking for employment and one moved from full time employment to part time employment, the mean number of days worked in the last 30 days went up from 4.0 to 5.5. Other notable changes include enrollment in school and enrollment in job training for distinct clients. No criminal justice involvement (arrests, jail, or prison) was reported in the 30 days before discharge. This is worth mentioning when considering that one client was in prison in the 30 days before admission and another client had been arrested and spent time in jail in the 30 days before treatment. One client who had visited an emergency room in the 30 days prior to admission did not return in the 30 days prior to discharge. Similarly, one client who had been hospitalized in the 30 days prior to admission was not re-hospitalized in the 30 days prior to discharge. In total, the number of clients reporting medical problems in the last 30 days dropped from 37.5% (*n *= 3) to 12.5% (*n *= 1) amongst those completing treatment. The three that had indicated medical problems in the 30 days before admission went from a mean of 4.375 days with medical problems to zero. One person discontinued use of psychiatric medication. Reiman [19] reported that many medical cannabis users sought marijuana as a less debilitating method of controlling psychiatric difficulties than traditional psychiatric medications. Further research into treatment outcomes of medical marijuana users might offer further insight into those findings.

Table 3 offers a comparison between medical marijuana using clients and the control group. Some of the data from Table 1 is repeated in Table 3 to highlight the areas where differences are apparent. Successful completion or satisfactory progress at discharge was 28.1 percentage points higher in the MM group than in the Non-MM group, while successful completion alone was 30.7 percentage points higher. The MM group stayed in treatment for at least four months at twice the rate as the Non-MM group (76.9% [*n *= 10] to 37.7% [*n *= 55]).

**Table 3:** Medical Marijuana Client (MM) and Non-Medical Marijuana (Non-MM) Client Outcomes

	Medical Marijuana Clients		Non-Medical Marijuana Clients	
	
	%	*n*	%	*n*
**Completed tx or made satisfactory progress at discharge**	69.2%	9	41.1%	60
**Completed tx**	61.5%	8	30.8%	45
**Unsatisfactory discharge**	30.8%	4	58.9%	86
**Received at least 4 months of tx**	76.9%	10	37.7%	55
**Primary drug use - no change**^ab^	37.5%	3	8.6%	5
**Primary drug use - reduction**^ab^	12.5%	1	5.2%	3
**Primary drug use - increase**^ab^	25.0%	2	13.8%	8
**Employed at discharge**^ac^	50%	3	33.9%	20
**Enrolled in school**^a^	12.5%	1	8.5%	5
**Enrolled in job training**^a^	12.5%	1	0.0%	0
**Any criminal justice involvement in last 30 days**^a^	0.0%	0	1.7%	1
**Arrested in last 30 days**^a^	0.0%	0	1.7%	1
**Jailed in last 30 days**^a^	0.0%	0	1.7%	1
**ER visit in last 30 days**^a^	0.0%	0	3.4%	2
**Hospitalized in last 30 days**^a^	0.0%	0	3.4%	2
**Medical problems reported in last 30 days**^a^	12.5%	1	8.5%	5
**MH diagnosis**^a^	50.0%	3	22.0%	13
**MH medication**^a^	25.0%	2	23.7%	14

a = For Medical Marijuana clients this only includes those who completed treatment. For Non-Medical Marijuana Clients this only includes a completed "AOD treatment services set" (a matching admission and discharge-excluding administrative discharges).

b = Thirty day use prior to admission compared to 30 day use prior to discharge.

c = Includes Full-time and Part-time, excludes Not In Labor Force.

84.6% (*n *= 11) of the MM group and 71.8% (*n *= 112) of the Non-MM group reported methamphetamine as a primary or secondary drug. This suggests that methamphetamine use was at least equally problematic in the MM cohort. 100% (*n *= 8) of the MM group reported marijuana as a primary or secondary drug of use at admission, while only 75% (*n *= 117) of the Non-MM group did so. Though not particularly relevant as it only represents one client, alcohol use was reported by 7.7% of the MM group as a primary or secondary drug, while 25% (*n *= 39) of the Non-MM group reported alcohol as a primary or secondary drug.

CalOMS records changes in drug-using behavior as "abstinence," "increase," "reduction," or "no change." This can lead to confusion in data interpretation. Abstinence is defined as no drug use at all in the 30 days before admission nor in the 30 days before discharge. The categories of "increase," "reduction," and "no change" only relate to those who were actively using alcohol or other drugs in the 30 day period before treatment admission. "Abstinence" numbers are 25% (*n *= 2) for the MM group and 72.4% (*n *= 42) for the Non-MM group, suggesting that many more of the Non-MM group were abstinent before treatment ever began. Amongst those that reported drug use at admission, the MM group showed a greater percentage of clients who reported an increase in number of days of primary drug use (25% [*n *= 2] to 13.8% [*n *= 8]). However, this group shows a proportionately greater percentage of clients who reported a *reduction *in primary drug use in the 30 days before discharge (12.5% [*n *= 1] to 5.2% [*n *= 3]). This seemingly contrary outcome (higher level of increase and higher level of reduction) is possible because of the smaller number of MM clients who were abstinent before admission. The MM group also demonstrated a greater percentage of clients with *no change *in their drug use (37.5% [*n *= 3] to 8.6% [*n *= 5]). As a large percentage of the MM group reported marijuana as their primary drug of use, it ought not be surprising that they continued to use marijuana regularly or even increased it. Since CalOMS data are presented in aggregate, it is not possible to determine the number of days of methamphetamine use in the 30 days prior to discharge amongst the Non-MM group. However, 26.5% (*n *= 9) of the Non-MM group reported some level of methamphetamine use at discharge, compared to none for the MM methamphetamine users.

A higher percentage of the MM group demonstrated preferred outcomes in relation to employment, school enrollment, job training enrollment, criminal justice involvement, ER visits, and hospitalizations, but the *n*s are too small to warrant significant attention. In fact, at the .05 level the small sample size of the MM group prevents meaningful statistical analysis altogether. If similar results were found in a proportionately larger sample size, the following differences likely would have been significant: treatment completion, at least 4 months of treatment, employed at discharge, and alcohol use at discharge. Clearly more research is warranted to answer questions about treatment effects raised here.

## Discussion

### Limitations

This research project is notably limited. Primarily, MM group members were identified by persons working in the treatment setting, not through official documentation. Unfortunately, California does not require treatment providers to indicate the medical marijuana status of a client when CalOMS data is reported. MM group identities were confirmed on multiple occasions and by more than one program staff member (though the identities were not shared with the researcher).

Aside from the small sample size, data gaps presented the other main limitation. CalOMS data for the control group is presented in aggregate, while the data for the MM group is much richer. This allows for more accurate representation of treatment outcomes for the MM group, but hinders the rigor of comparisons. So, for example, CalOMS reports client characteristics and treatment outcomes for all treatment episodes (service counts), while the MM group is only for the most recent treatment episode (unduplicated individuals). This means that CalOMS includes data on clients who moved in and out of treatment on multiple occasions. Also, CalOMS reports data on categories of discharge besides treatment completion, while the MM group only reports discharge information for treatment completers. Since the MM group *n*s were subtracted from the CalOMS reports for the entire county, this potentially included duplicated clients. There were 53 cases of "completed treatment" in the Non-MM group. The data comparing admission indicators to discharge indicators for this group included 67 cases. This suggests that discharge data were reported for 14 people who did not successfully complete treatment.

Another limitation in the study relates to its quasi-experimental nature. The quantity of marijuana used by members of the MM group is unknown, as are other important factors including frequency of use, potency of product, level of contamination, and method of ingestion. So, for example, while the bulk of marijuana consumed in the United States is produced in Mexico [24] it is more likely that the marijuana used in this study was secured from a regional source owing to the setting's geography. The American College of Physicians notes, "examining the effects of smoked marijuana can be difficult because the absorption and efficacy of THC on symptom relief is dependent on subject familiarity with smoking and inhaling. Experienced smokers are more competent at self-titrating to get the desired results. Thus, smoking behavior is not easily quantified or replicated" ([8], p. 35).

Cannabidiol (CBD) content is as important for ascertaining the effect of marijuana use as tetrahyrdocannabinol (THC). The lack of illness specific data limits the study's ability to draw powerful conclusions about marijuana's potential in addictions treatment. We know, for example, that CBD has some anti-psychotic and anti-anxiety properties [25-27]. Yet the percentage of clients who used medical marijuana for psychiatric difficulties rather than, for example, chronic pain is unknown. The data does indicate that 50% (*n *= 3) of MM group treatment completers had a mental health diagnosis compared to 22% (*n *= 13) of the Non-MM group.

### Suggestions for further research, practice, and policy

Expanded data collection is necessary while the "natural experiment" of authorized marijuana use continues in California. A very simple policy change, adding an additional question (i.e., Are you an authorized medical marijuana user? Yes/No) to the State of California's Outcomes Measurement System (CalOMS), would make rigorous data analysis possible by significantly increasing sample size. Clearly there are other questions related to marijuana use that would aid in any research project as well, such as frequency of use, potency of product, method of ingestion, and medical condition for which marijuana has been recommended. Though treatment clients currently participate in a lengthy interview at admission that generates data points for client characteristics, demographics, and patterns of behavior, introduction of too many additional questions would likely prevent requisite legislative action.

Sample size could be increased by involving additional counties at a higher level of engagement than that described here. Medical marijuana users themselves could also be recruited to participate in the research (for examples of medical marijuana user surveys see [19,28,29].

Most importantly, the study described here demonstrates a beginning methodology for determining medical marijuana's effects on substance abuse treatment outcomes. Research *can *be done even within legal and ethical constraints posed by cannabis research.

## Concluding considerations

The American College of Physicians position paper on "Supporting Research into the Therapeutic Role of Marijuana" references marijuana's analgesic qualities [8] while other sources address marijuana's potential in the context of mental illnesses, anorexia, nausea, and muscle spasticity [7,9-13]. How the findings described here relate to other studies on marijuana's potential as a therapeutic aid remains inconclusive. It is clear, however, that cannabis use did not compromise substance abuse treatment amongst the medical marijuana using group. In fact, medical marijuana users seemed to fare equal to or better than non-medical marijuana users in every important outcome category.

Movement from more harmful to less harmful drugs is an improvement worthy of consideration by treatment providers and policymakers. The economic cost of alcohol use in California has been estimated at $38 billion [30]. Add to this the harm to individuals, families, communities, and society from methamphetamine, heroin, and cocaine, and a justification can be made for medical marijuana in addictions treatment as a harm reduction practice. As long as marijuana use is not associated with poorer outcomes, then replacing other drug use with marijuana may lead to social and economic savings.

There are differences in public and professional perceptions about marijuana use. Thirty-two percent of Americans believe that addiction to marijuana is a danger to society [31]. However, the Institute of Medicine is quite clear in saying, "Marijuana has not been proven to be the cause or even the most serious predictor of serious drug abuse" ([7], p. 10). Marijuana dependence may very well be problematic, but the Institute of Medicine also concluded "compared with alcohol, tobacco, and several prescription medications, marijuana's abuse potential appears relatively small and certainly within manageable limits for patients under the care of a physician" (p. 58). Further research on marijuana's effects on treatment outcomes can help address the disparity in disciplinary perceptions and decision-making.

Hardly pro-marijuana lobbies, the National Institute on Drug Abuse, the Office of National Drug Control Strategy, and the State of California's Little Hoover Commission on California State Government Organization and Economy all make recommendations about substance abuse treatment services that are consistent with studying the potential for medical marijuana use in addictions care.

For at least a decade the National Institute on Drug Abuse has maintained that drug addiction is a brain disease [32]. California's Compassionate Use Act of 1996 (Section 11362.5 of California's Health and Safety Code) is equally clear that people "have the right to obtain and use marijuana for medical purposes where that medical use is deemed appropriate and has been recommended by a physician who has determined that the person's health would benefit from the use of marijuana in the treatment of cancer, anorexia, AIDS, chronic pain, spasticity, glaucoma, arthritis, migraine, *or any other illness for which marijuana provides relief*" (emphasis added). Expanding the evidence-base for effective addiction treatments through a variety of treatment protocols continues to be worthy of attention from research and clinical communities.

While it may sound contrarian to suggest that the federal government's *National Drug Control Strategy *might support research into the potential therapeutic effect of marijuana on problematic use of other drugs, the document emphasizes "the need for customized strategies that include behavioral therapies, medication, and consideration of other mental and physical illnesses" ([24], p. 31). Considering marijuana in a medicinal context, the research described here offers a novel customized strategy. The *National Drug Control Strategy *goes on to note, "Experience with methamphetamine abusers has shown that recovery can be achieved by focusing on sobriety, pharmacological intervention for any associated depression and anxiety that appear with sobriety, and the establishment of routines" (p. 31). Marijuana has already shown therapeutic potential for anxiety symptoms [10]. Just as anti-depressant medications are used in substance abuse treatment, marijuana may show promise as an additional pharmacological intervention for methamphetamine users, if the data presented here are replicated in larger-scale studies.

California's Little Hoover Commission on California State Government Organization and Economy has studied the state's system of substance abuse treatment twice in the last five years [33,34]. In their most recent analysis, the commission concluded that "the state should transform programs for nonviolent drug offenders by tying funding to outcomes, requiring drug court models where appropriate, and requiring counties to tailor programs to offenders' individual risks and needs." Supporting the use of marijuana during treatment follows from this recommendation unless such use demonstrates poorer outcomes, which is not indicated in the research described here.

From the perspective of abstinence-only treatment, 30 day drug use at discharge may be a key measure of treatment success or failure. With 87.5% (*n *= 7) of the MM group having used marijuana in the 30 days before discharge, the question could certainly be asked whether the overwhelming percentage of successful treatment completions noted in Table 1 ought really be considered positive. Furthermore, those indicating marijuana use in the 30 days before discharge had used cannabis anywhere from 14-30 days. This is clearly not abstinence. However, marijuana was the *only *substance with reported use in the 30 days before discharge, including amongst those who had reported use of alcohol and methamphetamine previously. Social, health, and behavioral outcomes for the MM group did not appear to be any worse than the Non-MM group.

Drug abuse screening tools do not tend to focus on frequency or quantity of use as an indicator of drug-related problems, nor do the diagnostic criteria for substance abuse or substance dependence. If clinical, moral, and legal concerns about marijuana use during treatment are set aside, we are left with measurable outcomes as the only meaningful indicators of success. Preliminary findings presented here lay out a systematic methodology for examining marijuana's effect on treatment outcomes.

## Conflict of Interest Statement

The author has no financial or personal relationships with people or organizations that could inappropriately influence or bias this work, including employment, consultancies, stock ownership, honoraria, paid expert testimony, or patent applications/registrations. The Marijuana Policy Project provided a "Marijuana Research Grant" that supported the study without any contractual constraints or edits.
